# Spatial and temporal patterns of dengue infections in Timor-Leste, 2005–2013

**DOI:** 10.1186/s13071-017-2588-4

**Published:** 2018-01-04

**Authors:** Kinley Wangdi, Archie C. A. Clements, Tai Du, Susana Vaz Nery

**Affiliations:** 1 Research School of Population Health, The Australian National University, Canberra, Australia; 2 ANU Medical School, The Australian National University, Canberra, Australia

**Keywords:** Dengue, Timor-Leste, Spatial analysis, Bayesian analysis, Time series analysis

## Abstract

**Background:**

Dengue remains an important public health problem in Timor-Leste, with several major epidemics occurring over the last 10 years. The aim of this study was to identify dengue clusters at high geographical resolution and to determine the association between local environmental characteristics and the distribution and transmission of the disease.

**Methods:**

Notifications of dengue cases that occurred from January 2005 to December 2013 were obtained from the Ministry of Health, Timor-Leste. The population of each suco (the third-level administrative subdivision) was obtained from the Population and Housing Census 2010. Spatial autocorrelation in dengue incidence was explored using Moran’s I statistic, Local Indicators of Spatial Association (LISA), and the Getis-Ord statistics. A multivariate, Zero-Inflated, Poisson (ZIP) regression model was developed with a conditional autoregressive (CAR) prior structure, and with posterior parameters estimated using Bayesian Markov chain Monte Carlo (MCMC) simulation with Gibbs sampling.

**Results:**

The analysis used data from 3206 cases. Dengue incidence was highly seasonal with a large peak in January. Patients ≥ 14 years were found to be 74% [95% credible interval (CrI): 72–76%] less likely to be infected than those < 14 years, and females were 12% (95% CrI: 4–21%) more likely to suffer from dengue as compared to males. Dengue incidence increased by 0.7% (95% CrI: 0.6–0.8%) for a 1 °C increase in mean temperature; and 47% (95% CrI: 29–59%) for a 1 mm increase in precipitation. There was no significant residual spatial clustering after accounting for climate and demographic variables.

**Conclusions:**

Dengue incidence was highly seasonal and spatially clustered, with positive associations with temperature, precipitation and demographic factors. These factors explained the observed spatial heterogeneity of infection.

**Electronic supplementary material:**

The online version of this article (10.1186/s13071-017-2588-4) contains supplementary material, which is available to authorized users.

## Background

Dengue fever is caused by dengue virus (DENV) serotypes 1 to 4 [1]. Infection with the dengue virus can produce a wide spectrum of illness and severity, ranging from a mild, non-specific febrile syndrome to classic dengue fever (DF), and the severe forms of the disease, dengue haemorrhagic fever (DHF) and dengue shock syndrome (DSS) [2, 3].

Dengue viruses are transmitted to humans through the bite of infected female *Aedes* mosquitoes, mainly by *Aedes aegypti* and *Ae. albopictus* (tiger mosquito) [4, 5]. *Aedes aegypti*, is predominantly an urban vector [6, 7], while *Ae. albopictus* is found more often in peri-urban and rural environments [8]. *Aedes* mosquitoes commonly breed in artificial containers, like flower vases, old automobile tires, buckets and empty containers, in residential areas [6, 8–11].

Dengue fever (DF) is the most common mosquito-borne viral disease in the world, with an estimated 20,000 deaths occurring per year as a result of severe cases of dengue, 50–100 million people being infected each year and 2.5 billon people living in at risk areas [12–14]. In the 1970s only nine countries were affected by dengue epidemics; now epidemics are reported in more than 100 countries, making dengue an international public health problem with an increasing disease burden and an expanding geographical range [12, 13, 15]. The incidence of dengue has increased in South-East Asia, Africa, the Western Pacific, and the Americas [6]. Dengue transmission occurs in all countries of the World Health Organization (WHO) South-East Asia region (SEAR), except North Korea, accounting for up to two-thirds of the global burden [13, 14]. In Timor-Leste, dengue remains a major public health problem with frequent outbreaks [12].

Dengue prevention and control in Timor-Leste is implemented using the guidelines outlined in the Bi-regional Dengue Strategy (2008–2015) (WHO South-East Asia and Western Pacific regions) [16]. Dengue control in Timor-Leste involves a multi-pronged approach based on case management through early detection and diagnosis, vector control via spraying, education on prevention and surveillance [12]. Dengue is mostly diagnosed based on the clinical findings. Whilst current guidelines recommend the use of RDTs, they are not being widely used. Dengue cases are of obligatory notification and cases reported are collated by the department of epidemiological surveillance at the Ministry of Health.

In the regional neighbouring countries of Singapore, Australia, Vietnam and Cambodia, spatial epidemiological studies of dengue have identified social, local and environmental risk factors of the disease and provided evidence for more targeted interventions [17–20]. Despite the high costs and burden of the disease [21, 22], there have been few epidemiological or clinical studies of dengue in Timor-Leste [18, 23, 24]. However, one potential difference in epidemiology of dengue in Timor-Leste from other countries in the region is that Timor-Leste doesn’t have any large cities - the largest city is Dili with around 200,000 people. Other countries that are more densely populated with larger urban populations might experience more intense transmission, and cities that are more connected with other cities and regions through transportation networks might experience greater exchange of pathogens than Dili.

Using geographical information system (GIS) and a Bayesian statistical framework, the present study aims to quantify the spatio-temporal patterns of notified dengue incidence in Timor-Leste between 2005 and 2013, to identify dengue clusters in the country at a high geographical resolution (at the suco level, which is a low-level administrative subdivision) and to visualise smoothed patterns of dengue risk. The study also aimed to quantify the role of local environmental factors, such as temperature and precipitation, in influencing the distribution and dynamics of the disease.

## Methods

### Study area

The Democratic Republic of Timor-Leste occupies a land area of 15,007 km^2^ in the eastern part of the island of Timor and is located between 8.1°S and 9.5°S, and 125.0°E and 127.3°E, including the small enclave of Oecussi between 9.2°S and 9.5°S and 124.1°E and 124.5°E located in the western half of the island within West Timor [25]. The country is divided into 13 municipalities, 67 sub-municipalities and 442 sucos [26, 27]. The population of Timor-Leste in the 2015 census was 1,167,242 [26]. Dili, the capital city, is the only major population centre with over 200,000 people, with no other towns having more than 20,000 people. The majority of the population lives in rural areas and practises subsistence farming. About 42% of the population lives below the poverty line, with an estimate adult literacy rate of 60% [28]. Topographically, the country is mostly comprised of mountainous terrain (80%) surrounded by coastal swamp plains with no permanent rivers [29].

### Data collection

The data for this study were provided by the Ministry of Health, Timor-Leste and consisted of patient records containing 4546 notifications, classified as DF, suspected dengue cases, DHF, and DSS from January 2005 to December 2013. However, after data cleaning only 3206 cases were matched and assigned to current recognised sucos of residence (the spatial unit of analysis). For this analysis, cases were stratified into two age groups reflecting dengue risk: < 14 years (children) and ≥ 14 years (older adolescents and adults), based on the population breakdown of Timor-Leste Population and Housing Census 2010 [27]. The population growth was extrapolated from Population and Housing Census 2010 with annual population growth rate of 2.41 [27].

Long-term average annual and seasonal mean temperature and precipitation variables were created using data retrieved from WorldClim at 1 km spatial resolution. These layers were produced by using a thin-plate smoothing spline algorithm to interpolate data collected from global weather station sources between 1950 and 2000. The raster data were extracted and aggregated at the suco level using the centroid of suco. An electronic map of suco boundaries in shapefile format was obtained from Global Administrative Areas database (http://www.gadm.org/country). Administrative boundaries of sucos in Timor-Leste have changed over time and there were 456 sucos in the map downloaded from the website, while dengue data were obtained from 442 sucos. In this analysis, 456 spatial units were used after reconciling the dengue data to match the boundary map.

### Exploration of seasonal patterns and interannual patterns

The average monthly number of dengue cases was calculated from the full time-series (January 2005-December 2013). The time series of dengue incidence was decomposed using seasonal-trend decomposition based on locally weighted regression to show the seasonal pattern, interannual patterns and the residual variability. Seasonal-trend decomposition was selected to understand the variation in time series with seasonal and trend components. The time series data, the seasonal component, the trend component and the remainder component were denoted by *Y*_*t*_, *S*_*t*_, *T*_*t*_ and *R*_*t*_, respectively, for *t* = 1 to N. Then

$$ {Y}_t={S}_t+{T}_t+{R}_t $$

In the study, *Y*_*t*_ specifically stands for local dengue cases with logarithmic transformation, *S*_*t*_ is the additive seasonal component, *T*_*t*_ is the trend, and *R*_*t*_ is the “remainder component”; *t* is time in unit of month [30–32].

### Spatial autocorrelation analysis

Spatial autocorrelation was explored at a global scale using Moran’s I statistic and at a local scale using local indicators of spatial association (LISA), estimated using the Anselin Local Moran’s I statistic, and the Getis-Ord statistic. The global Moran’s I statistic was used to assess the presence and strength of spatial autocorrelation over the whole study area and to test the assumption of spatial independence in the implementation of the spatial pattern analysis. The LISA and the Getis-Ord statistics were used to detect local clustering dengue and to identify the locations of hot-spots. These analyses were conducted using tools provided in ArcGIS [33].

### Data analysis

Zero-inflated Poisson regression models were constructed in a Bayesian framework using the WinBUGS software, version 1.4.3 (Medical Research Council, Cambridge, UK). The first model (Model I) included temperature, precipitation, age (< 14 years and ≥ 14 years) and gender as explanatory variables, and an unstructured random effect for sucos; the second model (Model II) included the same explanatory variables and a spatially structured random effect; the final model (Model III), a convolution model, contained all of the components of the preceding two models.

The last model, which had as an outcome the observed counts of dengue, *Y*, for *i*^th^ suco (*i* = 1...456) in the *j*^th^ month (January 2005-December 2013), for the *k*^th^ age group and *l*^th^ sex group was structured as follows:

$$ P\left({Y}_{ij}={y}_{ij}\right)=\left\{{}_{\left(1-\omega \right){e}^{-\mu }{\mu}_{ij}^{y_{ij}}/{y}_{ij},{y}_{ij}>0;}^{\omega +1\ \left(1-\omega \right){e}^{-\mu },\kern0.75em {y}_{ij}=0}\right. $$

$$ {\displaystyle \begin{array}{c}{Y}_{ij kl}\sim \mathrm{Poisson}\ \left(\mu ijkl\right)\\ {}\log\ \left({\mu}_{ij kl}\right)=\log \left({\mathrm{E}}_{ij kl}\right)+{\theta}_{ij kl}\\ {}{\theta}_{ij kl}=\alpha +{\beta}_1\times {\mathrm{Age}}_k+{\beta}_2\times {Sex}_l+{\beta}_3\times {\mathrm{Precipitation}}_{ij}+{\beta}_4\times {\mathrm{Tempmeanx}}_{ij}+{\mathrm{u}}_i+{\mathrm{s}}_i\end{array}} $$

where E_*ijkl*_ is the expected number of cases (acting as an offset to control for population size) in suco *i*, month *j*, age group *k*, sex group *l* and θ_*ijkl*_ is the mean log relative risk (RR); α is the intercept, and *β*_*1*_*, β*_*2*_*, β*_*3*_*,* and *β*_*4*_ the coefficients for age (< 14 years reference), sex (male reference), precipitation, and mean temperature, respectively; u_i_ is the unstructured random effect (assumed to have a mean of zero and variance σ_u_^2^) and s_i_ is the spatially structured random effect (assumed to have a mean of zero and variance σ_s_^2^).

A conditional autoregressive (CAR) prior structure was used to model the spatially structured random effect. Spatial relationships between sucos were determined using an adjacency weights matrix. If two sucos shared a border, a weight of 1 was assigned, while if they did not, weight was 0. A flat prior distribution was specified for the intercept, whereas a normal prior distribution was used for the coefficients (mean of zero and precision, the inverse of variance, set at 0.0001). The priors for the precision of unstructured and spatially structured random effects (inverses of the variances shown above), were specified using non-informative gamma distributions with shape and scale parameters equal to 0.001.

An initial burn-in of 1000 iterations was run, and these iterations were discarded. Subsequent blocks of 20,000 iterations were run and examined for convergence. Convergence was assessed by visual inspection of posterior density and history plots, and occurred at approximately 100,000 iterations for each model. Ten thousand values from the posterior distributions of each model parameter were stored and summarised for the analysis using the posterior mean and 95% credible intervals (95% CrI). The deviance information criterion (DIC) was calculated for each model and used for model selection, where a lower DIC indicates a better-fitting, more parsimonious model. In all analyses, an α-level of 0.05 was adopted to indicate statistical significance (as indicated by 95% CrI for relative risks (RR) that excluded 1).

ArcMap software was used to generate maps of the spatial distribution of posterior means of the unstructured and structured random effects.

## Results

### Descriptive statistics

There were a total of 4546 cases recorded during the study period; 47.3% (2151) patients were female. Female children (< 14 years) made up 32.4% (1475) and male children made 36.1 (1639) of total cases. Most cases, 40.6% (1846), were classified as DHF, followed by 30.8% (1398) classified as dengue fever and 27.8% (1266) as suspected cases (Table 1). DSS was reported in less than 1 % of total cases (36). Most cases of DSS, 63.9% (23), were reported in male children < 14 years, followed by female children < 14 years 19.4% (7) (Table 1). Rapid diagnostic tests, including IgM and IgG, were only used in 46.4% (2045) and 46.8% (2133) of the total cases, respectively (Additional file 1: Table S1), and PCR was carried out in only nine patients (result not shown).

**Table 1:** Dengue cases stratified by age and sex

Sex	Age (yrs)	Clinical types of dengue				Total
		DF	DHF	DSS	SC	
Female	< 14	399 (28.5)	656 (35.5)	7 (19.4)	413 (32.6)	1475 (32.4)
	≥ 14	245 (17.5)	251 (13.6)	2 (5.6)	178 (14.1)	676 (14.9)
Male	< 14	490 (35.1)	668 (36.2)	23 (63.9)	458 36.2)	1639 (36.1)
	≥ 14	264 (18.9)	271 (14.7)	4 (11.1)	217 (17.1)	756 (16.6)
Total		1398 (38.7)	1846 (40.6)	36 (0.8)	1266 (27.8)	4546

*Abbreviations*: DF, dengue fever; DHF, dengue haemorrhage fever; DSS, dengue shock syndrome SC, suspected cases

**Fig. 1:**
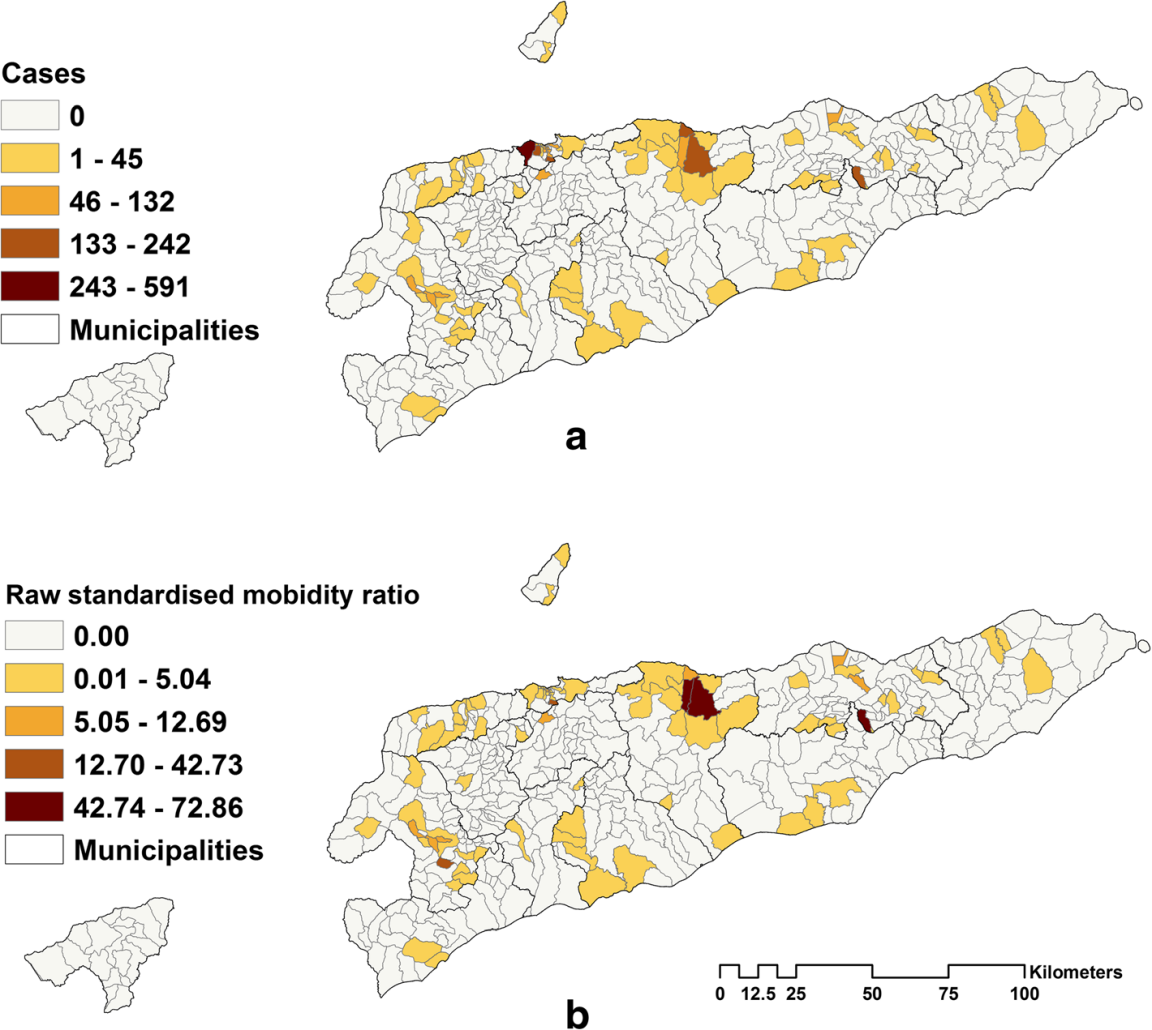
**a** Distribution of reported dengue cases by residence. **b** Raw standardised morbidity rations for dengue by sucos in Timor-Leste for the period 1 January 2005 to 31 December 2013

A total of 3206 notified cases of dengue infections (DF, DHF and DSS and suspected cases) for the period from 2005 to 2013 were successfully matched to location (place of residence of the patient), using the most recent administrative divisions of the country. The rest of the cases (*n* = 1320) could not be matched to residential sucos and were not considered further. Males made up 53.4% (1713) and children (< 14 years) made up 69.6% (2232) of the cases (Additional file 1: Table S2). The overall incidence was 3.4 and 5.8 cases per 10,000 person years at risk for adults and children, respectively. There was strong spatial variation in the dengue cases across sucos (Fig. 1). Only 83 of 442 sucos reported dengue cases, with the highest number of cases from Comoro suco followed by Tirilolo and Bairo Pite with 592 (18.5%), 242 (7.5%) and 235 (7.3%), respectively, all in Dili municipality. Twenty sucos reported only one case of dengue during the study period. Municipalities containing sucos with the most cases were Dili with 53.5% (1715) of cases, followed by Manatuto with 16.7% (535 cases) and Baucau with 14.2% (455 cases) (Table 2).

**Table 2:** Dengue cases stratified by municipalities

Municipality	No. of reporting sucos	Total cases	Percent
Aileu	1	53	1.7
Ainaro	2	2	0.1
Baucau	14	455	14.2
Bobonaro	12	353	11.0
Covalima	2	12	0.4
Dili	20	1715	53.5
Ermera	2	2	0.1
Lauteum	2	2	0.1
Liquica	8	33	1.0
Manatuto	12	535	16.7
Manufahi	5	23	0.7
Viqueque	3	21	0.7
Total	83	3206	100

### Time-series decompositions

**Fig. 2:**
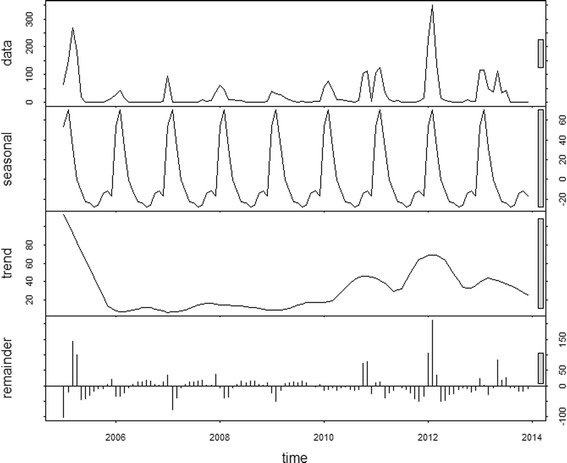
Decomposed dengue cases of Timor-Leste, 2005–2013

The time-series decompositions are shown in Fig. 2. A clear seasonal pattern is evident in the raw data. There are two seasonal peaks: a bigger peak usually in January, preceded by a smaller one in December. The inter-annual pattern showed a large peak in 2005 and three small peaks in 2011, 2012 and 2013, with lower incidence in the intervening and subsequent years (Fig. 2).

### Spatial autocorrelation analysis

**Fig. 3:**
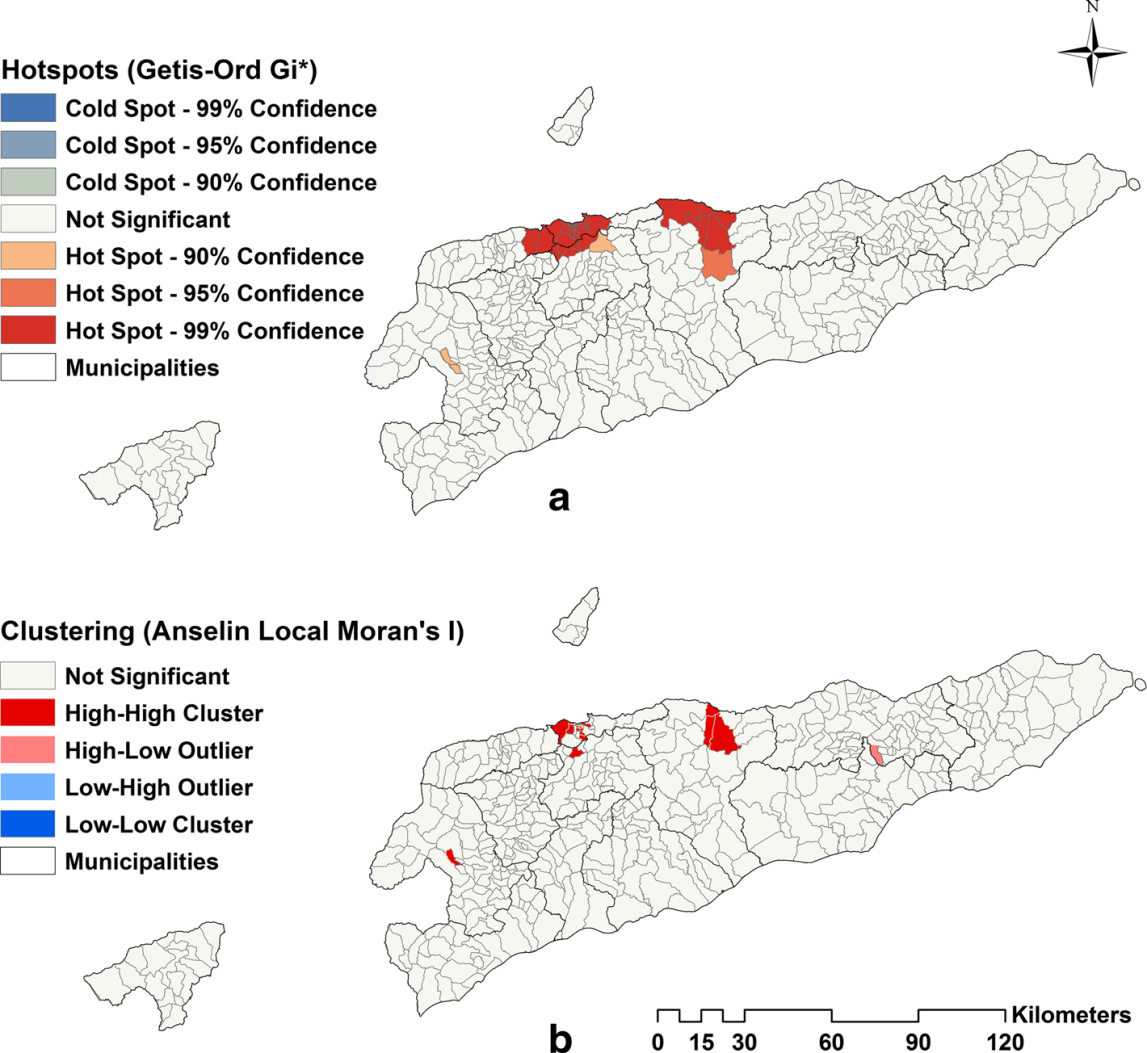
Spatial clustering of for dengue by sucos in Timor-Leste for the period 1 January 2005 to 31 December 2013 based on: the Getis-Ord Gi* statistics (**a**) and Local indicators of spatial association using Anselin Local Moran’s I statistic (**b**)

The global Moran’s index statistic for the dengue cases was 0.28 (*P*-value < 0.0001), indicating the presence of significant, positive spatial autocorrelation of dengue over the whole study area. Hotspot analysis using the Getis-Ord statistic (Fig. 3a) showed that 41 sucos were located in significant hotspots located in and around Dili and Manatuto. However, cluster analysis using LISA showed only 18 high-high clusters (Fig. 3b), also mainly located in and around Dili and Manatuto.

### Spatial Poisson regression analysis

As indicated by the low DIC value in Table 3, Model I (the model with an unstructured random effect) had a better fit than the models containing spatially structured random effects. This suggests that despite the presence of spatial autocorrelation for the dependant variables in the exploratory phase of the study, and as evidenced by the global and local Moran’s I statistic presented above, the inclusion of covariates resulted in no residual spatial autocorrelation, and thus the CAR random effects models were redundant. In other words, the covariates included in the models explained the spatial clustering evident in the dengue data.

**Table 3:** Regression coefficients, RRs and 95% CrI from Bayesian spatial and non-spatial models for dengue cases from 2005 to 2013

Model/variables	Coefficient, posterior mean (95% CrI)	RR, posterior mean (95% CrI)
Model I (unstructured)		
α (Intercept)	-14.09 (-16.1, -11.03)	
Children^a^	-1.343 (-1.429, -1.257)	0.26 (0.24, 0.285)
Sex^b^	0.111 (0.035, 0.187)	1.12 (1.036, 1.206)
Temperature mean (°C)	0.007 (0.006, 0.008)	1.007 (1.006, 1.008)
Precipitation (mm)	0.382 (0.258, 0.462)	1.47 (1.294, 1.587)
Omega	0.77 (0.753, 0.786)	
Heterogeneity		
Structured	–	–
Unstructured	0.068 (0.047, 0.094)	–
DIC	13,685.9^c^	
Model II (structured)		
α (Intercept)	-23.07 (-27.75, -15.0)	
Children^a^	-1.34 (-1.568, -1.118)	0.26 (0.208, 0.327)
Sex^b^	0.115 (-0.105, 0.34)	1.12 (0.9, 1.405)
Temperature mean (°C)	0.007 (0.004, 0.009)	1.01 (1.004, 1.009)
Precipitation (mm)	0.502 (0.244, 0.685)	1.65 (1.276, 1.984)
Omega	0.767 (0.735, 0.797)	
Heterogeneity		
Structured	0.02 (0.014, 0.028)	–
Unstructured	–	–
DIC	13,808.2	
Model III (structured and unstructured)		
α (Intercept)	-18.33 (-24.42, -7.246)	
Children^a^	-1.342 (-1.46, -1.225)	0.26 (0.232, 0.294)
Sex^b^	0.114 (−0.001, 0.232)	1.12 (0.999, 1.261)
Temperature mean (°C)	0.007 (0.005, 0.009)	1.01 (1.005, 1.009)
Precipitation (mm)	0.409 (0.023, 0.59)	1.51 (1.023, 1.804)
Omega	0.768 (0.747, 0.788)	
Heterogeneity		
Structured	0.067 (0.024, 0.155)	–
Unstructured	0.139 (0.072, 0.279)	–
DIC	13,709.5	

*Abbreviations*: CrI, credible interval; RR, relative risk; DIC, deviance information criterion

^a^Age < 14 years was reference

^b^Male sex was reference

^c^Best-fit model

**Fig. 4:**
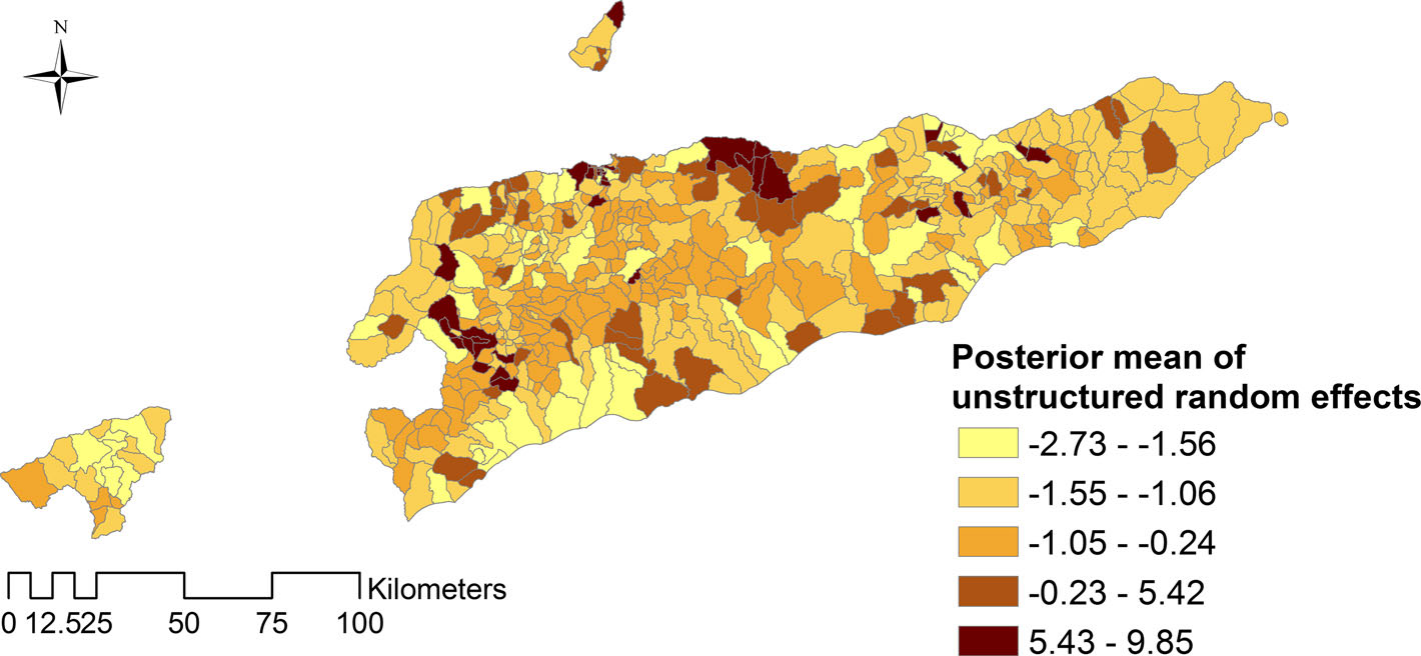
Spatial distribution of the posterior means of unstructured random effects for dengue in Timor-Leste in Model I

In the best-fit model (Model I), individuals aged ≥ 14 years were found to be 74% (95% CrI: 72–76%) less likely to have a dengue infection than children aged < 14 years, and females were 12% (95% CrI: 4–21%) more likely to suffer from dengue when compared to males. Dengue incidence increased by 0.7% (95% CrI: 0.6–0.8%) for a 1 °C increase in mean temperature; and 47% (95% CrI: 29–59%) for 1 mm increase in precipitation (Table 3). The maps of the posterior means of the spatially unstructured random effects demonstrated little evidence of spatial clustering after accounting for the model covariates (Fig. 4, Additional file 2: Figure S1; Additional file 3: Figure S2).

## Discussion

This is the first study to explore spatiotemporal patterns of dengue in Timor-Leste. There was no evidence of spatial clustering of dengue risk after accounting for the covariates, suggesting that variability in precipitation and temperature explain much of the spatiotemporal dynamics of the disease. This is similar to findings from studies in other parts of the world [34, 35]. However, it is possible that spatial clustering occurs at other scales, such as at the household level and during the outbreaks, because *Aedes* mosquitoes mostly breed in and around the house [36].

Dengue in Timor-Leste is highly seasonal, with peaks in cases occurring during the wettest and hottest months of the year (December-February). Similar findings have been reported in other studies that showed a strong seasonal pattern of dengue [17, 37]. The increase in dengue cases with increase in mean temperature and precipitation are likely to be associated with the vector dynamics [38, 39]. With the increase in temperature, the longevity of the *Aedes* mosquitoes increases, with the best survival temperatures occurring between 27 and 30 °C [40–44]. Higher temperatures also shorten the extrinsic incubation period of DENV within mosquitoes [45]. In places where there is water shortage, increased temperature leads to storage of water in containers, which in turn provides a breeding place for mosquitoes [46, 47]. Similarly, increased precipitation would facilitate vector population growth by providing water for mosquito breeding [38, 48].

Higher temperatures in Asia have been caused by strong *El Niño* cycle [49], suggesting that temperature variation might also play a role in interannual patterns of dengue. Whilst the time-series was not long enough to assess long-term temporal trends, the peaks in the latter part of the study period are consistent with reports of recent upward trends in dengue cases in a number of countries in SEAR [6, 50]. A number of plausible reasons are responsible for this surge in dengue cases, including population expansion and increasing migration from rural to urban regions where dengue risk is higher [51]. This was evident from dengue risk being spatially clustered in and around Dili and Manatuto. Dili, being the capital city of Timor-Leste, is confronted with a number of problems typical of urban areas in developing countries, such as increasing population through migration from rural areas and an unplanned expansion of the city. This could have led to substandard water and sanitation provision with improper disposal of waste, such as bottles and containers, which provide breeding places for *Aedes* mosquitoes.

Dengue is predominantly a childhood disease, with other studies reporting the risk of dengue decreasing with age [52–56]. This is plausible since *Aedes* mosquitoes breed in water containers placed in and around houses, where children are likely to spend most of their time [52]. This offers opportunities for repeated bites by mosquitoes and facilitates transmission of DENV. Additionally, adults are less likely than children to suffer from dengue infection because of acquired immunity that has developed as a result of dengue infection in childhood. [57]. The higher number of dengue cases observed in young children could also be because of higher notification rates; parents may be more likely to take their children to the health facility or hospital than they do themselves in the event of sickness.

Females were more likely to suffer from dengue than males, which might be explained by the fact that females are more likely to work in and around the home and are therefore at an increased risk of bites by *Aedes* mosquitoes. Similar findings were also reported in another study in Timor-Leste [24].

There are a number of limitations to this study. First, the dengue data were collected through passive surveillance of people with fever cases attending a health unit and, for the most part, being clinically diagnosed, which overlooks the contribution of asymptomatic dengue fever cases. Secondly, the quality of the dengue surveillance system may vary over time and between locations, as the awareness of dengue among medical professionals and public health workers may have increased as a result of the WHO’s initiatives to train and sensitize aforementioned health workers. It is possible that the national surveillance system operates more effectively in areas in and around the capital, and less effectively in remote areas. This might have contributed to the low estimated disease burden outside of Dili and Manatuto municipalities.

## Conclusions

In conclusion, dengue in Timor-Leste is highly seasonal with inter annual variations. Climatic factors including mean temperature and precipitation were important predictors of dengue cases. Significant inter-annual variability was found in disease risk. This calls for public health actions to mitigate future risks from climate change. In addition, children and females are at higher risk of dengue.

## Additional files

Additional file 1: Table S1. Results of two rapid test (IgM and IgG). **Table S2.** Stratified dengue cases by gender and age of Timor-Leste, 2005-2013. (DOCX 14 kb)

Additional file 2: Figure S1. Spatial distribution of the posterior means of random effects for dengue in Timor-Leste in Model III. **a** Spatially unstructured random effects. **b** Structured random effects. (TIFF 68692 kb)

Additional file 3: Figure S2. Spatial distribution of the posterior means of structured random effects for dengue in Timor-Leste in Model II. (TIFF 34339 kb)

## Abbreviations

AIC: Akaike’s information criterion
BIC: Bayesian information criterion
CAR: Conditional autoregressive
CrI: Credible interval
DENV: Dengue virus
DF: Dengue fever
DHF: Dengue haemorrhage fever
DIC: Deviance information criterion
DSS: Dengue shock syndrome
GIS: Geographical information system
LISA: Local Indicators of Spatial Association
MCMC: Markov chain Monte Carlo
RR: Relative risk
SC: Suspected cases
SEAR: South-East Asia region
SMR: Standardised morbidity ratios
WHO: World Health Organization
