# Supplementary material for: Spatial and temporal patterns of dengue infections in Timor-Leste, 2005–2013
Source: Parasit Vectors. 2018 Jan 4;11:9. doi: 10.1186/s13071-017-2588-4 (PMC5755460; doi:10.1186/s13071-017-2588-4)
Supplement: Supplementary file 1 — Results of two rapid test (IgM and IgG). Table S2. Stratified dengue cases by gender and age of Timor-Leste, 2005-2013. (DOCX 14 kb) [file 13071_2017_2588_MOESM1_ESM.docx]

Additional file 1: Table S1 Results of two rapid tests (IgM and IgG)

|  | | **Rapid IgM (%)** | | |  |
| --- | --- | --- | --- | --- | --- |
|  |  | Missing | Negative | Positive | Total |
| **Rapid IgG (%)** | Missing | 2,334 (93.3) | 125 (5.0) | 43 (1.7) | 2,502 (55.0) |
|  | Negative | 5 (0.4) | 930 (65.9) | 476 (33.7) | 1,411 (32.5) |
|  | Positive | 75 (11.8) | 217 (43.2) | 342 (53.9) | 634 (13.9) |
|  | Total | 2,414 (53.1) | 1,272 (27.9) | 861 (18.9) | **4,547** |

Additional file 1: Table S2 Stratified dengue cases by gender and age of Timor-Leste, 2005-2013

| **Year** | **Children (<14years)** | | **Adults (≥14 years)** | | **Total** |
| --- | --- | --- | --- | --- | --- |
|  | Male (%) | Female (%) | Male (%) | Female (%) |  |
| **2005** | 314 (44.2) | 212 (29.8) | 98 (13.8) | 87 (12.2) | 711 |
| **2006** | 39 (43.8) | 33 (37.1) | 10 (11.2) | 7 (7.9) | 89 |
| **2007** | 56 (37.6) | 57 (38.3) | 17 (11.4) | 19 (12.8) | 149 |
| **2008** | 57 (41) | 45 (32.4) | 16 (11.5) | 21 (14.4) | 146 |
| **2009** | 45 (34.6) | 37 (28.5) | 21 (16.2) | 27 (20.8) | 130 |
| **2010** | 165 (38.1) | 131 (30.3) | 64 (14.8) | 73 (16.9) | 433 |
| **2011** | 87 (28.8) | 90 (29.8) | 58 (19.2) | 67 (22.2) | 302 |
| **2012** | 271 (36.1) | 249 (33.2) | 124 (16.5) | 107 (14.2) | 751 |
| **2013** | 199 (39.6) | 145 (28.9) | 72 (14.3) | 86 (17.1) | 502 |
