# Supplementary figures and images for: Spatial and temporal patterns of dengue infections in Timor-Leste, 2005–2013
Source: Parasit Vectors. 2018 Jan 4;11:9. doi: 10.1186/s13071-017-2588-4 (PMC5755460; doi:10.1186/s13071-017-2588-4)

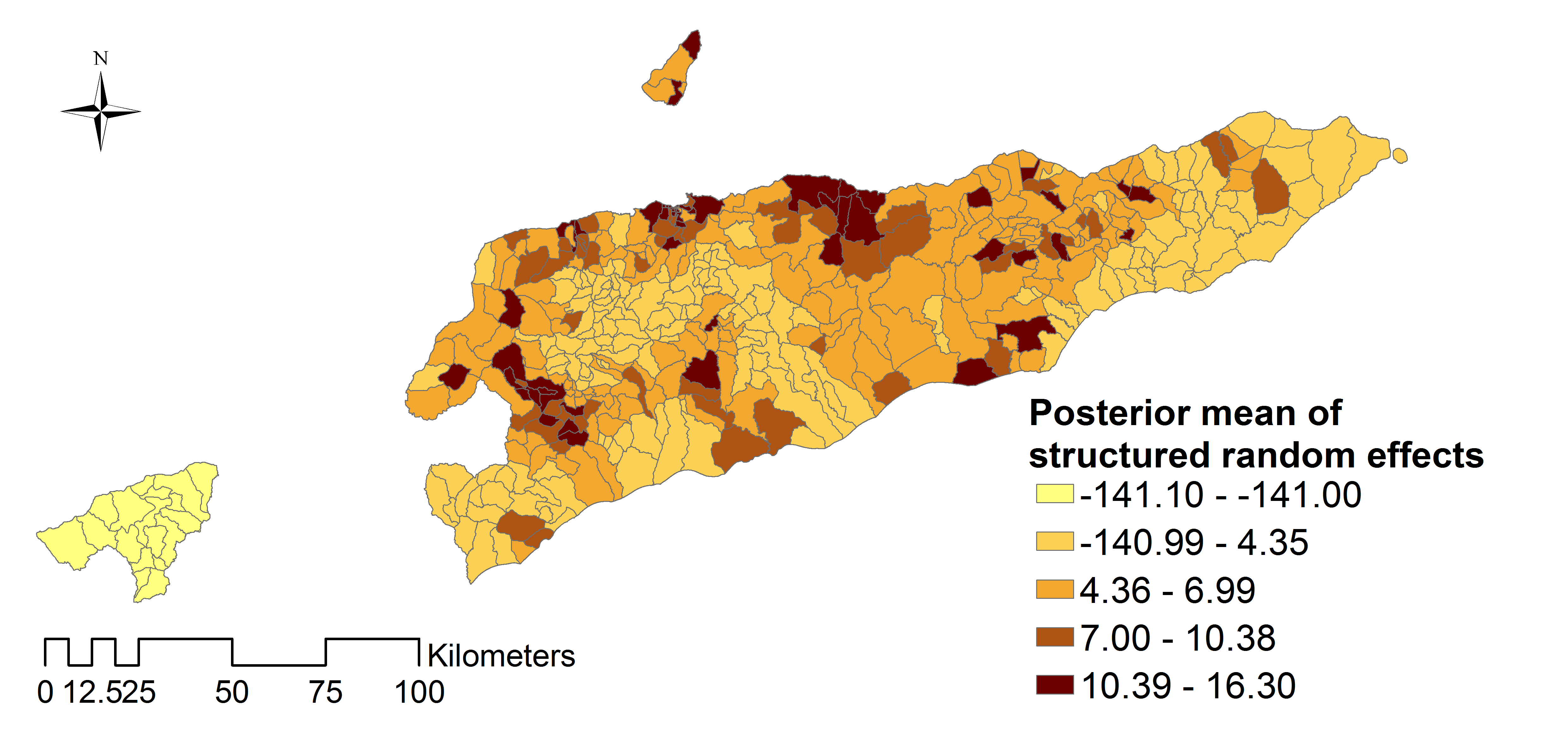

Supplement: Supplementary file 3 — Spatial distribution of the posterior means of structured random effects for dengue in Timor-Leste in Model II. (TIFF 34339 kb) [file 13071_2017_2588_MOESM3_ESM.tif]
